# Solamargine Inhibits the Development of Hypopharyngeal Squamous Cell Carcinoma by Decreasing LncRNA *HOXA11-As* Expression

**DOI:** 10.3389/fphar.2022.887387

**Published:** 2022-07-12

**Authors:** Ying Meng, Mengli Jin, Dai Yuan, Yicheng Zhao, Xiangri Kong, Xuerui Guo, Xingye Wang, Juan Hou, Bingmei Wang, Wu Song, Yong Tang

**Affiliations:** ^1^ College of Clinical Medicine, Changchun University of Chinese Medicine, Changchun, China; ^2^ College of Integrated Chinese and Western Medicine, College of Rehabilitation, Changchun University of Chinese Medicine, Changchun, China; ^3^ Center of Infections Diseases and Pathogen Biology, Key Laboratory of Organ Regeneration and Transplantation of the Ministry of Education, First Hospital of Jilin University, Changchun, China; ^4^ Affiliated Hospital to Changchun University of Chinese Medicine, Changchun University of Chinese Medicine, Changchun, China; ^5^ School of Pharmacy, Jilin University, Changchun, China

**Keywords:** solamargine, hypopharyngeal squamous cell carcinoma, HOXA11-AS, miR-155, c-Myc

## Abstract

Hypopharyngeal squamous cell carcinoma (HSCC) is one of the high mortality cancers with a poor prognosis, which is driving the development of new chemotherapeutic agents. We identified the anticancer effects of a natural compound, solamargine (SM), on FaDU cells and explored its mechanism in terms of non-coding RNA. It was observed that SM inhibited the proliferation of FaDU cells with an IC_50_ of 5.17 μM. High-throughput sequencing data revealed that lncRNA *HOXA11-AS* was significantly downregulated in cells co-incubated with SM. Further assays demonstrated that SM-induced downregulation of lncRNA *HOXA11-AS* showed important implications for apoptosis. Given the properties of *HOXA11-AS* as a *miR-155* sponge, we further confirmed that SM upregulated the expression of *miR-155* in FaDU cells. *C-Myc* is a transcription factor that regulates cell differentiation and apoptosis, whose mRNA is considered to be targeted by *miR-155*. We showed that *c-Myc* expression was downregulated by SM and accompanied by increased apoptosis, which was consistent with the findings of transcriptome sequencing. Furthermore, SM administration suppressed xenograft tumor growth in a xenograft mouse model *in vivo.* In the light of the aforementioned findings, our results suggested that SM downregulated the expression of *HOXA11-AS*, which in turn induces apoptosis by downregulating *c-Myc* in FaDU, providing evidence for the anticancer effect of SM on HSCC and uncovering the effect of SM on non-coding RNAs as, at least partly, a mechanism of action.

## Introduction

Hypopharyngeal cancer (HPC) is a subtype of head and neck cancer, which has a poor prognosis than other head and neck cancers, with an overall survival rate of only 20% (Hoffman et al., 1997; Iwae et al., 2017). In the United States, the annual incidence of HPC is approximately 3,000 cases per year, with the majority of patients in advanced/late stages (Kuo et al., 2014). Hypopharyngeal squamous cell carcinoma (HSCC) is a predominant histotype of HPC that accounts for 95% of the total HPC types (Gang et al., 2022). There are significant regional variations in the epidemiological distribution of HSCC, with the highest incidence in South–Central Asia (Bray et al., 2018), which may be related to smoking, alcohol consumption, and dietary habits (Kwon and Miles, 2019). What is more worrying is that HSCC is prone to recurrence, and nearly 50% of patients recur within 1 year of diagnosis, often accompanied by metastatic (Hall et al., 2008).

LncRNA, a class of RNA transcripts greater than 200 bp length, has been a hot topic in tumor research (Peng et al., 2017). It is involved in the regulation of cancer development (Ijaz et al., 2018), including cell proliferation, migration, invasion, and apoptosis. *HOXA11-AS* (NCRNA 00076), the antisense strand of the *HOXA11* gene, regulates the growth and metastasis of cancer cells and is currently considered a cancer biomarker and therapeutic target (Xue et al., 2018). Recently, *HOXA11-AS* has been recognized to hold application potential as a circulating biomarker for early detection of HSCC, which promoted HSCC development by sponge to tumor suppressor *miR-155* (Xu et al., 2020).

Natural products have long been an essential source of anticancer agents (Rastelli et al., 2020). Solamargine (SM), a steroidal alkaloid glycoside derived from plants of the Solanum species such as *Solanum nigrum*, has been considered to carry the anticancer activity for decades (Kalalinia and Karimi-Sani, 2017). SM has shown potent anticancer effects exhibiting a very low IC_50_, against a variety of cancers including liver cancer (Sani et al., 2015), lung cancer (Liang et al., 2004), breast cancer (Shiu et al., 2007), melanoma, prostate cancer (Xiang et al., 2016), colon cancer (Rastelli et al., 2020), and cervical cancer (Kalalinia and Karimi-Sani, 2017). One study comparing the sensitivity of SM to conventional chemotherapy agents on lung cancer cells showed that SM was the most sensitive of these chemotherapy agents including paclitaxel and cisplatin (Liu et al., 2004). In another study on human melanoma cancer cells, SM was shown to exert anticancer effects with a low impact on normal and benign WM35 cells, suggesting its selectivity for primary melanoma cells WM239 (Al Sinani et al., 2016). These characteristics of SM prompted us to explore its anticancer activity against HSCC and the involvement of non-protein-coding RNAs (ncRNA).

In the present study, we preliminarily evaluated the effect of SM on FaDU cell phenotypes. High-throughput sequencing was utilized to examine the regulation of lncRNAs by SM treatment (Booton and Lindsay, 2014). According to the vital role of lncRNA *HOXA11-AS* in HSCC and our data obtained from high-throughput sequencing, we revealed the involvement of *HOXA11-AS* in the inhibitory effect of SM on the proliferation of FaDU cells. These findings may contribute to the development of SM as an anticancer compound for HSCC applications.

## Materials and Methods

### Cell Culture

The FaDU cell lines were obtained from the Affiliated Hospital of Changchun University of Chinese Medicine. FaDU cells were cultured in RPMI-1640 (Servicebio, Wuhan, China) supplemented with 10% fetal bovine serum (Servicebio, Wuhan, China), and 1% penicillin and streptomycin (Servicebio, Wuhan, China). The cells were cultured at 37°C in a 5% CO_2_ incubator.

### MTT Assay

The MTT assay was applied to determine the effect of SM on FaDU cell viability (Jin et al., 2014). FaDU cells were seeded into 96-well culture plates at 8,000 cells per well. The adherent cells were treated with 2 and 5 µM SM for 24, 48, and 72 h. After that, 10 μl of MTT solution (5 mg/ml) was added to each well and incubated at 37°C for 4 h. Then, the MTT solution was removed, and DMSO was used to dissolve formazan. The optical density (OD_490 nm_) value was detected using a microplate reader (Multiskan FC, Thermo Fisher) at 490 nm.

### EdU Staining

The cell proliferation ability was detected by 5-ethynyl-2′-deoxyuridine (EdU) staining (Kazda et al., 2016). FaDU cells were plated into 24-well culture plates at a density of 1 × 10^4^ cells/well. After 24 h, an equal volume of culture medium containing EdU (10 μM) was added to the cells and incubated for 2 h at 37°C, 5% CO_2_. Cells were fixed and permeabilized following EdU kit (Beyotime, Beijing, China) according to the manufacturer’s instructions. The cell nuclei were labeled with Hoechst 33342 stain. The results were photographed using an inverted fluorescence microscope, and the values of EdU-positive were analyzed by ImageJ software.

### Live/Dead Cell Staining

Live/dead cell staining was performed to evaluate the effects of SM on FaDU cell viability. FaDU cells were cultured in 24-well plates at 5 × 10^4^ cells per well. After treatment with SM (2 μM and 5 µM) for 24 h, cells were stained following the live/dead cell staining kit instructions (Beyotime, Beijing, China), at room temperature, away from light, for 30 min. Later, the cells were washed with phosphate-buffered saline (PBS). Fluorescence images were captured by inverted fluorescence microscopy (Leica, Wetzlar, Germany).

### Transwell Assay

To examine the invasive ability of FaDU cells, we performed a transwell assay. In total, 1 × 10^5^ cells per well were seeded in the upper compartment of Matrigel-coated invasion chambers in FBS-free media. Next, 500 µl RPMI-1640 medium containing 10% FBS was added to the lower chamber. After 24 h, migrated cells were fixed with 4% paraformaldehyde, stained with crystal violet, and counted with microscopy and ImageJ software.

### Clone Formation Assay

For colony formation assay, FaDU cells were seeded in six-well cell culture plates at 2,000 cells per well overnight. Then, the medium was replaced by the culture medium containing 2 µM SM for 10 days. Every 3 days, the culture medium in the wells was replaced. All cells were washed with PBS, fixed with 4% paraformaldehyde, and stained with crystal violet (Beyotime, Beijing, China). The images of the colony were photographed using the camera, and ImageJ was used to count them.

### Wound Healing Assay

The migration activity of FaDU cells was detected using wound healing assay. FaDU cells were seeded at 6 × 10^5^ cells per well of a six-well plate. When the cells reached 90% confluence, a 200-μL pipette tip was used to produce scratch wounds. Then, cells were treated with 2 and 5 μM SM. The area of the FaDU migrating into the wound was recorded with microscopy at time points of 0, 24, and 48 h and analyzed with ImageJ software.

### Cell Cycle and Apoptosis Assay

FaDU cells were cultured at a density of 8 × 10^5^ cells per well in six-well plates. Cells were then treated for 24 h with 2 and 5 μM SM. For assay of the cell cycle, Cell Cycle and Apoptosis Analysis Kit (Beyotime, Shanghai, China) was applied. Cells were stained according to the kit instructions after being fixed overnight at 4°C in 70% ethanol. Detection of experimental results was carried out using flow cytometry. For the assay of cell apoptosis, Annexin V-FITC apoptosis detection kit (Beyotime, Shanghai, China) was applied. The drug-treated cells were collected and stained according to the kit instructions. Detection of experimental results was carried out using flow cytometry and analysis with FlowJo software.

### LncRNA-Seq and Data Analysis

FaDU cells were cultured in 10-cm Petri dishes. After attachment, cells were treated with 2 and 5 μM SM for 24 h. LncRNA sequencing was performed on total RNA from cells isolated with TRIzol solution. After removal of ribosomal RNA, the next-generation sequencing (NGS) library was constructed, and sequencing of the constructed libraries was performed with Illumina NovaSeq. Trim_galore (0.6.4) was used to remove spliced sequences, and sequences with QCs less than 25 from the raw sequencing data were read to obtain clean data. Clean data were aligned to the human genome (hg38) using HISAT2 (2.1.0). Transcripts were quantified and annotated using feature counts (1.3.3) to obtain counts for each transcript. Transcripts were normalized using DESeq2, and KEGG enrichment analysis was performed for differentially expressed genes. The KEGG enrichment analysis of differentially expressed genes was performed using the R package clusterProfiler. Tumor tissues refer to head and neck tumor tissues, and normal tissues refer to the tissues of patients without head and neck cancer. Gene expression data from patients with HPC and clinical data were downloaded from TCGA. The R package “survival” was used to calculate the survival rate of patients and construct Kaplan–Meier curves.

### qRT-PCR

FaDU cells were cultured in 6-cm Petri dishes. After attachment, cells were treated with 2 and 5 μM SM for 24 h. Total RNA was extracted using TRIzol (Invitrogen). Then, using the SweScript All-in-One First-Strand cDNA Synthesis SuperMix for qPCR (One-Step gDNA Remover) (Servicebio, Wuhan, China) to synthesize cDNA according to the instructions. In addition, real-time fluorescent quantitative PCR was performed on a PikoReal 96 Real-Time PCR system using 2 × Fast SYBR Green qPCR Master Mix (None ROX) (Servicebio, Wuhan, China). GAPDH was used as an internal control, and the relative gene expression was calculated with the 2^−ΔΔCt^ method.

### SiRNA Transfection

To further investigate the molecular mechanism of SM-regulated lncRNA in HSCC, we performed the knockdown assay. FaDU cells were cultured in 10-cm dishes. When cell confluency met approximately 75%, cells were transfected with *si-HOXA11-AS* or si-NC using Lipofectamine™ 3000 (Invitrogen). The expression level of lncRNA HOXA11-AS in transfected FaDU was verified by qPCR. The siRNA oligonucleotide sequences were 5′-AAUGAGAGAGUGUAAUCAAGAUUdTdT-3’ (*si-HOXA11-AS*) and 5′-UCGUCAACGUUGCAUUGCGAUAUdTdT-3’ (si-NC).

### Dual-Luciferase Reporter Gene Assay

The luciferase reporter plasmid (lncRNA HOXA11-AS-WT or lncRNA HOXA11-AS-MUT) and miR-155 mimics were transfected into 293T cells, respectively. After 24 h of transfection, cells were lysed. Following the instructions of the Dual-Luciferase Reporter Gene Assay Kit (Beyotime, Shanghai, China), we set up different groups. After adding the firefly luciferase reaction solution, we detected the firefly luciferase fluorescence intensity using a microplate reader (Multiskan FC, Thermo Fisher). Then, we added the Renilla luciferase reaction solution and detected the Renilla luciferase fluorescence intensity.

### Immunoblotting Assay

Drug-treated FaDU cells were collected, and the proteins were isolated by lysis in RIPA buffer. Subsequently, protein concentrations were determined using a BCA protein reagent assay kit (Beyotime, Shanghai, China). Next, 12% sodium dodecyl sulfate-polyacrylamide gel electrophoresis (SDS-PAGE) was performed. Primary antibodies against c-Myc, P53, and anti-β-actin (Proteintech, 60004-1-Ig) were used overnight at 4°C. The secondary antibody was a goat anti-mouse HRP secondary antibody. The Western blot image was captured by Fusion FX Imaging systems (VILBER Lourmat, Collégien, France), and densitometric analysis was performed by ImageJ.

### Xenograft Tumor Model

Six-week-old male BALB/c mice right axillae were shaved. FaDU cells (2 × 10^6^) were injected subcutaneously into the right axilla of the mice. After 1 week of inoculation, 10 mice were randomly divided into two groups; the control group was injected with saline, and the SM group was injected with 10 mg/kg/d SM. Mice in each group were injected once daily for 30 days. The weight of the mice was recorded every 3 days. At the end of the treatment, the mice were sacrificed, the transplanted tumor was removed, and the tumor volume was recorded and weighed.

### Statistical Analysis

Data were statistically analyzed with GraphPad Prism 9.3.0. All experiments were performed in triplicate. Data are expressed as mean ± SD, and a two-sided *p* < 0.05 was considered significant.

## Results

### SM Inhibits FaDU Cell Proliferative and Migration Ability

**FIGURE 1:**
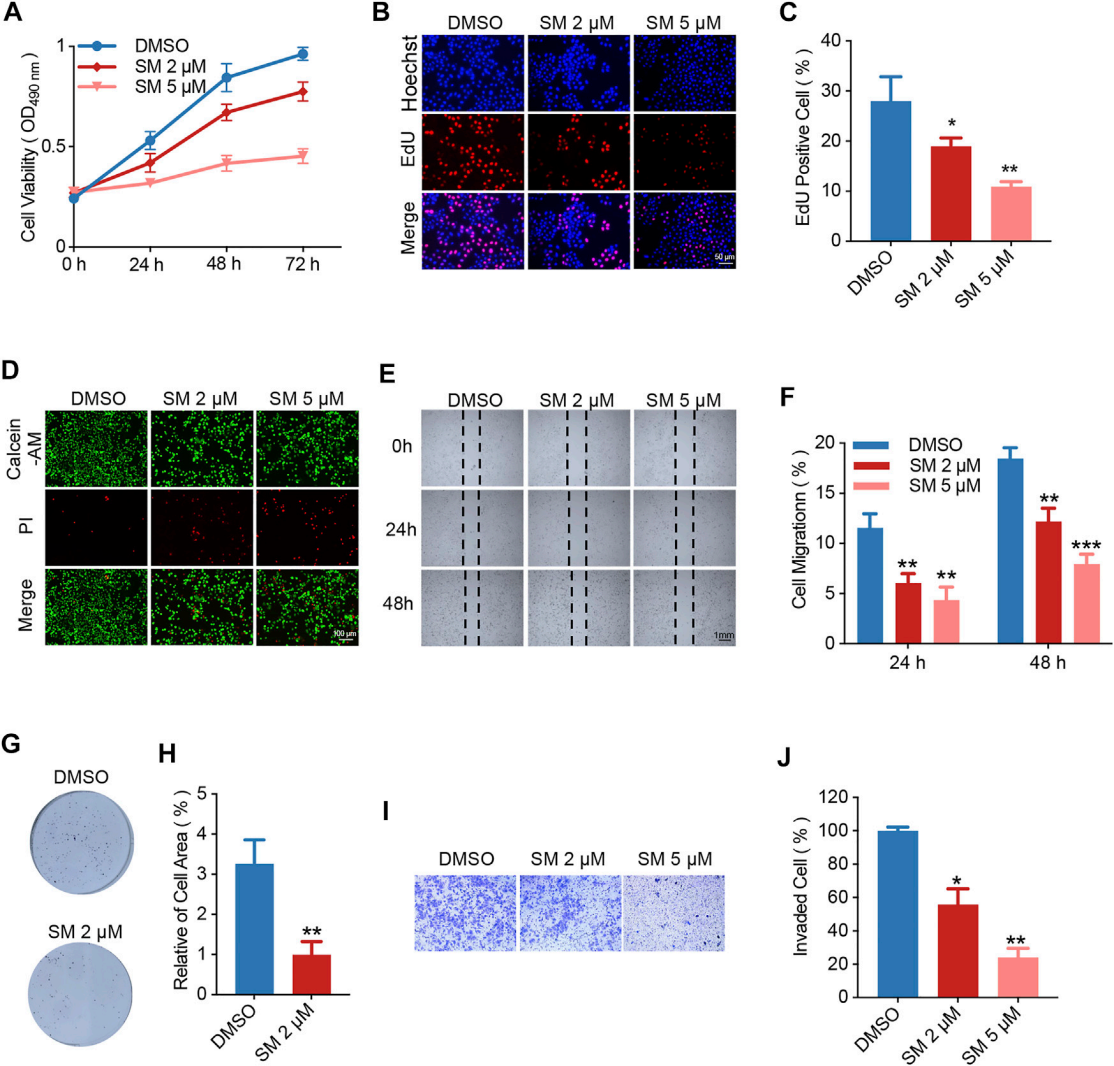
SM Inhibits FaDU cell proliferative and migration ability. **(A)** Cell viability of FaDU cells treated with DMSO and 2 and 5 μM SM was analyzed by MTT assay at 0, 24, 48, and 72 h (*n* = 5). **(B,C)** EdU/Hoechst staining of FaDU cells treated with SM (2 and 5 μM) for 48 h. The ratio of EdU^+^ cells calculated using ImageJ software was used to characterize cell proliferation (*n* = 3). **(D)** Calcein AM/PI staining of FaDU cells treated with SM (2 and 5 μM) for 48 h. **(E,F)** Cell migration of FaDU cells was tested by wound healing assay after treatment with DMSO and 2 and 5 μM SM, and the ratio was calculated by ImageJ software. **(G,H)** Colony formation of FaDU cells was performed with DMSO and 2 and 5 μM SM. Relative of cell area statistic with ImageJ software. **(I,J)** Cell invasion was analyzed using the transwell assay of FaDU cells treated with DMSO and 2 and 5 μM SM. The relative migration rate was calculated with ImageJ software. ^*^(*p* < 0.05), ^**^(*p* < 0.01), and ^***^(*p* < 0.001) indicate statistically significant differences.

In order to clarify the cytotoxic effect of SM on FaDU cells, the MTT assay was carried out on FaDU cells treated with SM (2 and 5 μM) for 72 h. The results showed that SM significantly inhibited the cell viability in a dose-dependent and time-dependent manner, and 5 μM SM inhibited cell proliferation by 50.68% for 48 h in MTT assays (Figure 1A). The IC_50_ (48 h) value of SM was 5.17 μM. EdU staining showed that novel proliferating cells were significantly reduced after SM treatment, as exhibited by the red fluorescence of EdU-positive cells attenuated in SM-treated FaDU cells (Figures 1B,C). The cytotoxic effect of SM on FaDU cells was then demonstrated by Calcein-AM/propidium iodide (PI) staining, as shown by an increase in red fluorescence of dead cells labeled by PI (Figure 1D). To evaluate the effect of SM on the migratory capacity of cells, wound healing assay was performed. We observed that SM inhibited cell migration and that SM (2 and 5 μM) increased the area of the wound compared to the DMSO group, indicating that cell migration was stifled by SM (Figures 1E,F). Clonogenic assays were performed to examine the impact of SM on the tumorigenicity of FaDU cells. The results showed that 2 μM SM greatly reduced the number of cell clones compared with the DMSO group, whereas FaDU cells treated with 5 μM had almost no clones visible after 10 days of incubation (data not shown) (Figures 1G,H). To examine the effect of SM on the invasion and migration ability of FaDU, the transwell assay was conducted. The results showed that SM (2 and 5 μM, for 24 h) had a significant inhibitory effect on the invasion ability of FaDU (Figures 1I,J). In summary, we verified for the first time the killing effect of SM on FaDU cells as well as its effect on cell migration and invasion capacity.

### SM Mediates Apoptosis and Cycle Arrest in FaDU Cells

**FIGURE 2:**
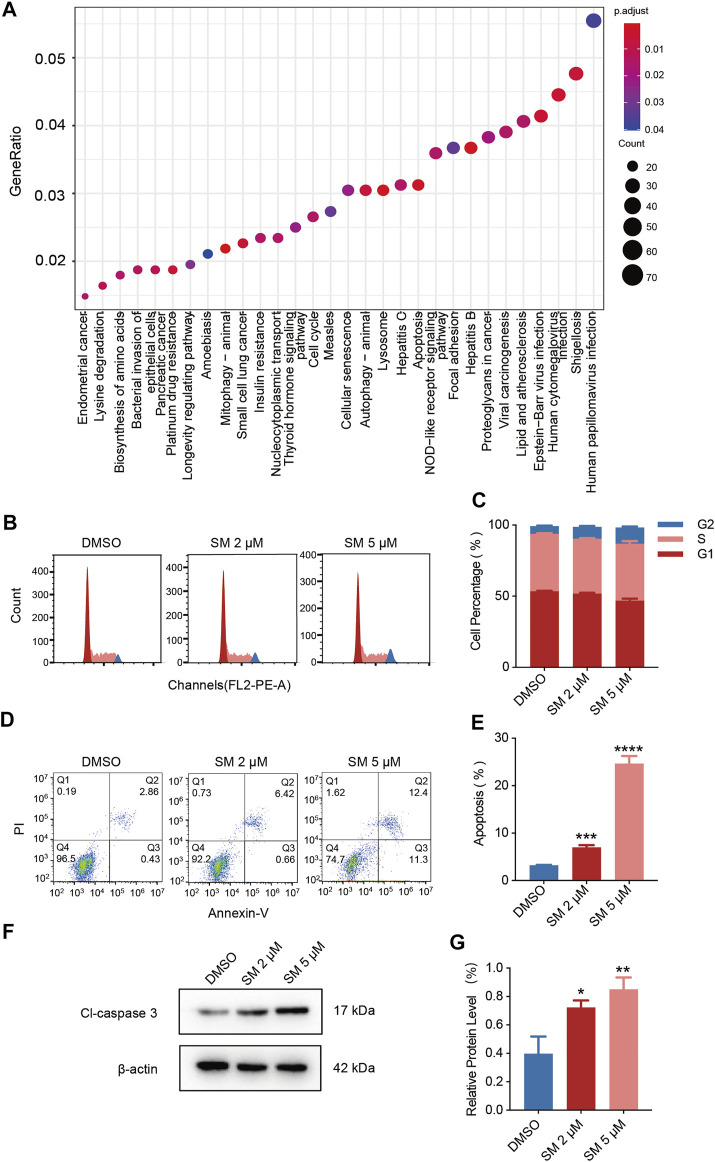
SM mediates apoptosis and cycle arrest in FaDU cells. **(A)** Enriched KEGG pathways of differentially expressed mRNAs in FaDU cells after 5 μM SM treatment. *p*-value < 0.05. **(B,C)** Cell cycle assay was examined by flow cytometry in FaDU cells treated with DMSO and 2 and 5 μM SM. The result was analyzed using FlowJo software. **(D,E)** Apoptosis of FaDU cells treated with DMSO and 2 and 5 μM SM was analyzed using flow cytometry, and the total apoptosis proportion was calculated by FlowJo software. **(F,G)** Effect of SM on apoptosis determined by the level of the cleaved caspase-3 protein. ^*^(*p* < 0.05), ^**^(*p* < 0.01), ^***^(*p* < 0.001), and ^****^(*p* < 0.0001) indicate statistically significant differences.

To uncover the potential mechanism of SM, we performed transcriptome sequencing and lncRNA high-throughput sequencing of 5 μM SM-treated FaDU. KEGG and GO enrichment analyses were performed on transcriptome sequencing results, in which a multitude of altered cellular pathways, apoptosis, and cycle arrest were floated. In the following assays, we examined the effect of SM on apoptosis and cycle in FaDU cells by flow cytometry (Figure 2A). Flow cytometry results for the cell cycle showed that the proportion of cells in the G2 phase increased and that of G1 phase cells decreased in SM-treated FaDU cells, indicating that SM resulted in the G2 phase block in FaDU cells (Figures 2B,C). The proportion of apoptosis in FaDU cells was increased by SM treatment, in which the proportion of early apoptosis increased (Q3) from 0.41 ± 0.02% to 11.73 ± 0.91% while that of late apoptosis (Q2) increased from 2.80 ± 0.06% to 12.93 ± 0.45% in FaDU cells treated with 5 μM SM for 48 h (Figures 2D,E).

### SM Suppresses the Expression of LncRNA *HOXA11-As,* Which Is Responsible for the Proliferation of FaDU Cells

**FIGURE 3:**
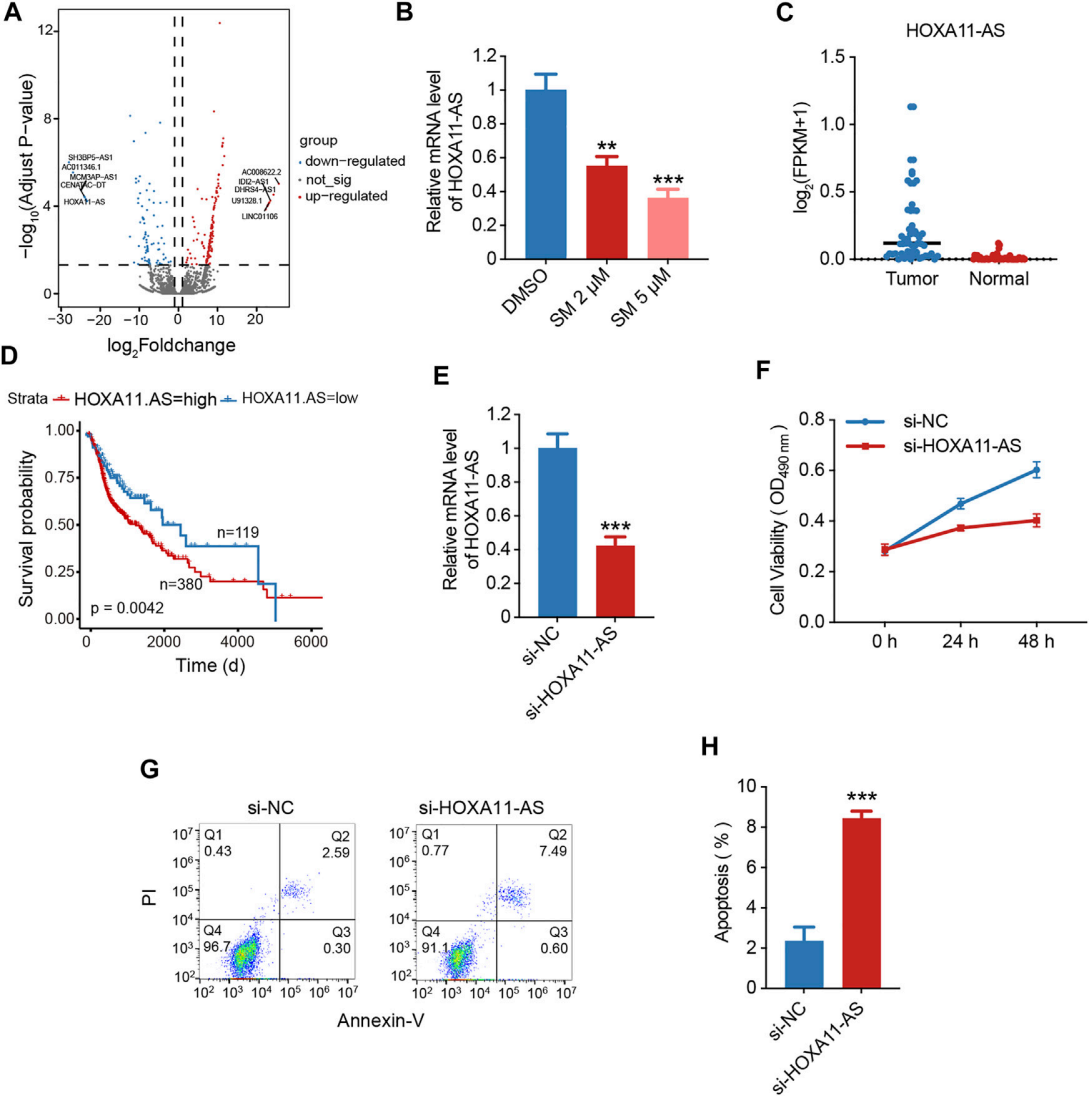
SM suppresses the expression of lncRNA *HOXA11-AS,* which is responsible for the proliferation of FaDU cells. **(A)** Volcano plot shows differentially expressed lncRNAs in FaDU cells treated with DMSO and 5 μM SM. Blue dots represent the downregulated lncRNAs, while red dots represent the upregulated ones. **(B)** Relative expression of lncRNA *HOXA11-AS* was analyzed by RT-PCR after DMSO and 2 and 5 μM SM treatment in FaDU cells. **(C)** Expression of *HOXA1-AS* in head and neck tumor tissue and in normal tissue. The cut-off value for low and high *HOXA11-AS* expression was 0.03143. **(D)** Impact of lncRNA *HOXA11-AS* expression levels on survival of patients with hypopharyngeal cancer in TCGA database. **(E)** Relative expression of lncRNA *HOXA11-AS* in FaDU cells transfected with *si-Nc* and *si-HOXA11-AS* was detected by RT-PCR. **(F)** Cell viability of FaDU cells transfected with *si-Nc* and *si-HOXA11-AS* was analyzed by MTT assay at 0, 24, and 48 h (*n* = 5). **(G,H)** Apoptosis of FaDU cells transfected with *si-Nc* and *si-HOXA11-AS* was analyzed by flow cytometry, and the total apoptotic percentage was calculated using FlowJo software. ^**^(*p* < 0.01) and ^***^(*p* < 0.001) indicated statistically significant differences.

Regarding lncRNA sequencing, the sequencing data showed that 244 differentially expressed lncRNAs were found in the SM-treated FaDU cells compared to that of untreated cells, of which 94 lncRNAs were downregulated and 150 lncRNAs were upregulated. From the significantly downregulated lncRNA, we focused on the *HOXA1-AS* (Figure 3A). Previous studies have suggested that *HOXA11-AS* is located in the *HOXA* gene cluster and its aberrant expression plays an important role in the development of various cancers such as non-small cell lung cancer, hepatocellular carcinoma, gastric cancer, and breast cancer (Wei et al., 2020). We performed the qRT-PCR assay for validation of lncRNA high-throughput sequencing findings, showing that SM was able to reduce *HOXA1-AS* expression in FaDU (Figure 3B). According to The Cancer Genome Atlas (TCGA) database, it was shown that the expression of *HOXA1-AS* was higher in head and neck tumor tissue than that in normal tissue (Figure 3C). Furthermore, patients with hypopharyngeal cancer with high *HOXA1-AS* expression were associated with lower survival rates (*p* = 0.0042), suggesting that the lncRNA *HOXA1-AS* represents a key point in the regulation of HSCC development (Figure 3D). The following experiments were carried out to identify the effect of *HOXA1-AS* on the proliferation and apoptosis of the HSCC cell line FaDU. As shown in Figure 3E, FaDU cells were transfected with *si-HOXA11-AS.* After transfection, qRT-PCR detection showed that the expression inhibition rate of *HOXA11-AS* in FaDU cells was 57.65%. To verify the role of *HOXA11-AS* in FaDU cell viability, we performed MTT assays on transfected cells, which showed a decrease in cell viability after 48 h of transfection, with 33.24% reduction compared to the control group (Figure 3F). We performed the cell counting test and confirmed consistent results that inhibition of *HOXA11-AS* led to a decrease in cell proliferation (Figure 3G). In order to verify the effect of *HOXA11-AS* on apoptosis in FaDU cells, apoptosis was measured by flow cytometry using Annexin-V/PI double staining and showed that transfection with *si-HOXA11-AS* caused an increase in the proportion of apoptotic cells, from 2.37 ± 0.56% to 8.46 ± 0.2% in the *si-HOXA11-AS* group compared to the si-NC group (Figure 3H). The aforementioned results suggested that downregulation of lncRNA *HOXA11-AS* by SM may be a prominent mechanism for SM to inhibit FaDU cell proliferation.

### SM Regulates *miR-155*-Induced Apoptosis by Reducing LncRNA *HOXA1-AS* Expression

**FIGURE 4:**
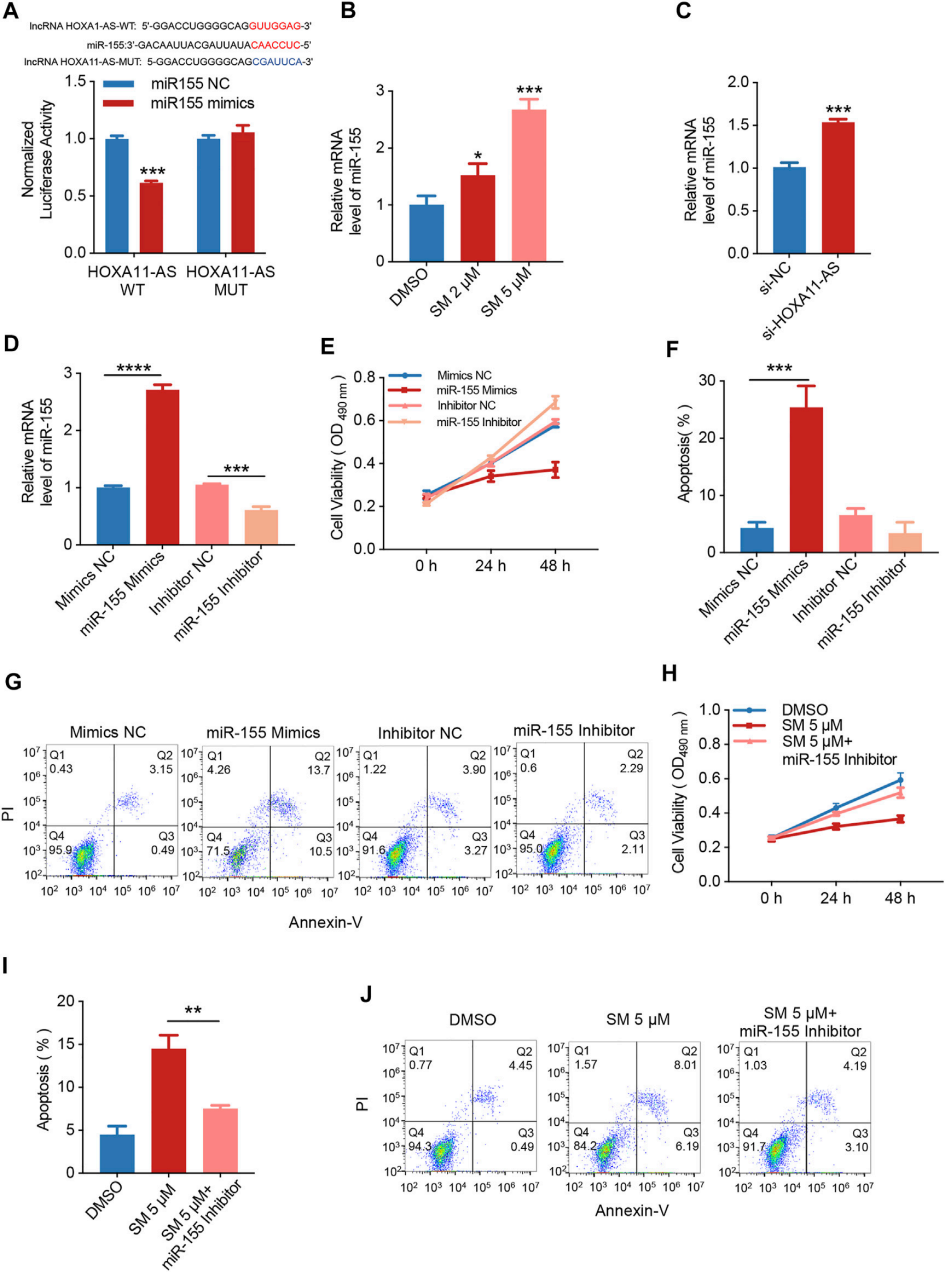
SM regulates *miR-155*-induced apoptosis by reducing lncRNA *HOXA1-AS* expression. **(A)** Relative luciferase activity of 293 cells co-transfected with *miR-155* NC or *miR-155* mimics and the wild-type or mutated lncRNA *HOXA11-AS*. **(B)** Relative expression of *miR-155* was analyzed by RT-PCR after DMSO and 2 and 5 μM SM treatment in FaDU cells. **(C)** Relative expression of *miR-155* analyzed by RT-PCR transfected with *si-NC* or *si-HOXA11-AS* in FaDU cells. **(D)** Relative expression of *miR-155* in FaDU cells was analyzed by RT-PCR after being transfected with the *miR-155* mimic and inhibitor. **(E)** Cell viability of FaDU cells transfected with the *miR-15*5 mimic and inhibitor was analyzed by MTT assay at 0, 24, and 48 h (*n* = 5). **(F,G)** Apoptosis of FaDU cells transfected with the *miR-155* mimic and inhibitor was analyzed by flow cytometry, and the total apoptotic percentage was calculated using FlowJo software. **(H)** Cell viability of FaDU cells transfected with the *miR-155* inhibitor and treated with 5 μM SM was analyzed by MTT assay at 0, 24, and 48 h (*n* = 5). **(I,J)** Apoptosis of FaDU cells transfected with the *miR-155* inhibitor and treated with 5 μM SM was analyzed using flow cytometry, and the total apoptosis proportion was calculated by FlowJo software. ^*^(*p* < 0.05), ^**^(*p* < 0.01), ^***^(*p* < 0.001), and ^****^(*p* < 0.0001) indicate statistically significant differences.

The base pairing pattern diagram is shown in Figure 4A to predict lncRNA *HOXA11-AS* targeting *miR-155*. This finding is supported by a previous study that lncRNA *HOXA11-AS* promoted the proliferation of HSCC through the sponge *miR-155* (Xu et al., 2020). The results of the dual-luciferase reporter gene assay confirmed that lncRNA *HOXA11-AS* could bind to *miR-155* and negatively relate with the expression of *miR-155*. In addition, the wiggle plot of lncRNA *HOXA11-AS* showed that the RNA-seq signal covers the potential *miR-155* binding site (Supplementary Figure S1A). To investigate whether *miR-155* is involved in the regulation of SM inhibition of FaDU cell proliferation, the qRT-PCR assay was carried out, showing *miR-155* was elevated by 1.65-fold when treated with 5 μM SM for 48 h compared to control cells (Figure 4B). The relative expression of *miR-155* was analyzed by RT-PCR transfected with si-NC or *si-HOXA11-AS* in FaDU cells (Figure 4C). FaDU cells were transfected with *miR-155* mimics and inhibitors to alter *miR-155* expression for subsequent assays with the purpose of testing the effect of differences in the *miR-155* expression on the proliferation and apoptosis of FaDU cells (Figure 4D). Data of MTT assays demonstrated that cells transfected with *miR-155* inhibitors displayed higher cell viability than the negative control, while lower viable in *miR-155* mimics-transfected cells (Figure 4E). In the apoptosis assay, a 21.07% increase in the apoptosis proportion was observed in cells transfected with *miR-155* mimics (Figures 4F,G). These results suggested that increasing *miR-155* expression suppressed cell viability and induced apoptosis. Furthermore, we evaluated the proliferation and apoptosis effects of 5 μM SM treatment on cells transfected with the *miR-155* inhibitor which showed a 41.58% increase in cell viability compared to SM treatment alone (Figure 4H), as well as a 44.29% decrease in apoptosis proportion (Figures 4I,J). The proliferation-inhibiting and apoptosis-inducing effects of SM on FaDU cells were attenuated by the inhibition of *miR-155* expression, suggesting that *miR-155* may be involved in the mechanism of SM combating FaDU cells. Moreover, SM could significantly upregulate the expression level of the cleaved caspase-3 protein (Figures 2F,G). According to the fact that lncRNA *HOXA1-AS* sponges *miR-155*, combined with the regulatory effect of SM on the expression of lncRNA *HOXA1-AS* and *miR-155*, we considered that downregulation of lncRNA *HOXA1-AS* and upregulation of *miR-155* were, at least partly, involved in inhibition of the proliferation of FaDU cells and apoptosis induction of SM.

### SM Induces Apoptosis via LncRNA *HOXA11-AS*/*miR-155*/*c-Myc*/p53

**FIGURE 5:**
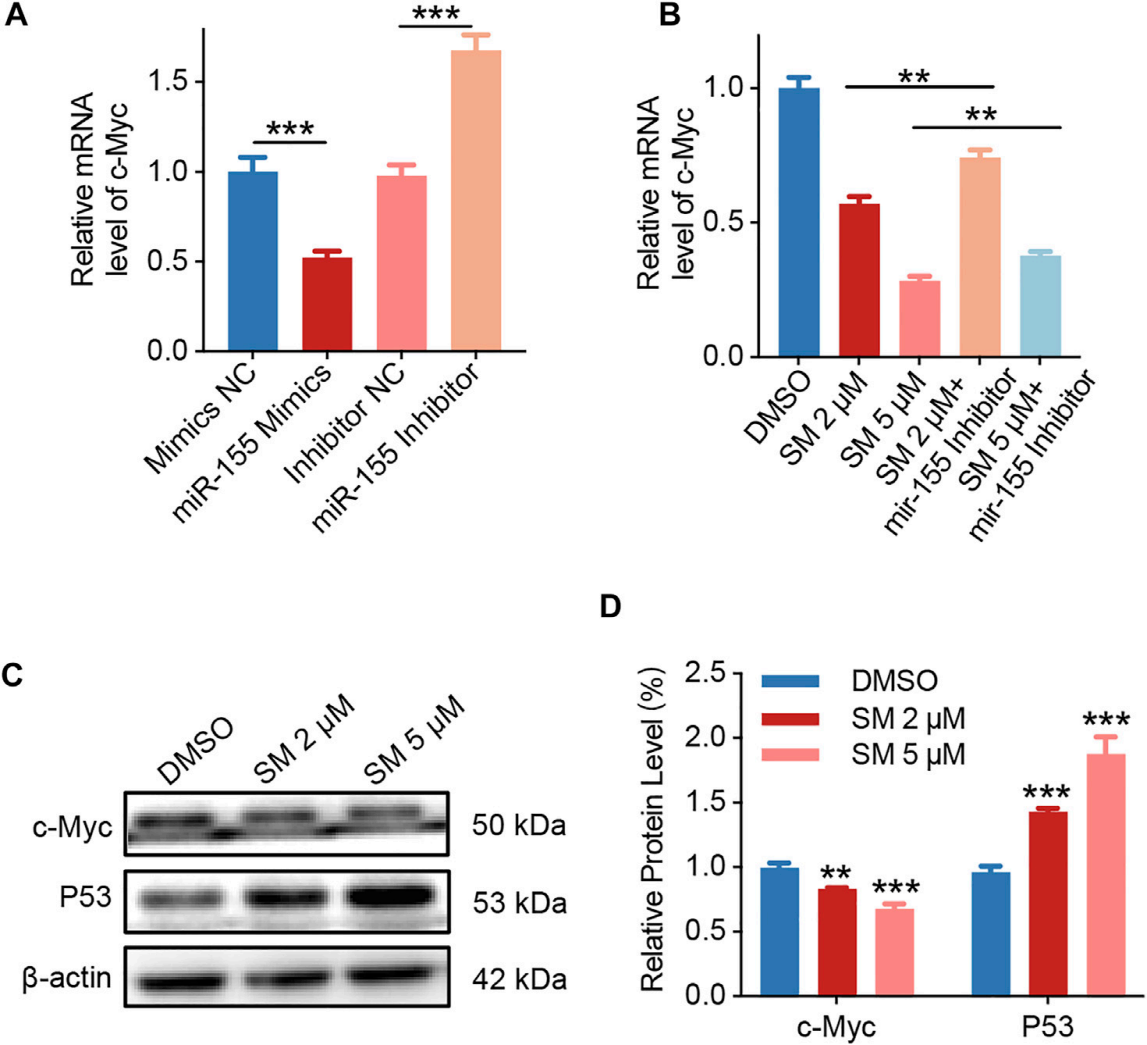
SM induces apoptosis via lncRNA *HOXA11-AS*/*miR-155*/*c-Myc.* **(A)** Relative expression of *c-Myc* in FaDU cells was analyzed by RT-PCR after being transfected with the *miR-155* mimic and inhibitor. **(B)** Relative expression of *c-Myc* in FaDU cells was analyzed by RT-PCR after being transfected with the *miR-155* inhibitor and treated with 2 and 5 μM SM. **(C,D)** Immunoblotting assay analysis of c-Myc and P53 in FaDU cells treated with DMSO and 2 and 5 μM SM. The grayscale values of the strips were analyzed with ImageJ software. ^**^(*p* < 0.01) and ^***^(*p* < 0.001) indicate statistically significant differences.

Studies have shown that the transcription factor *c-Myc*, which regulates cell apoptosis, is an important target gene for *miR-155*. The base pairing pattern diagram of *miR-155* and *c-Myc* sequence is shown in Supplementary Figure S1B. Furthermore, the qRT-PCR results showed that *miR-155* mimics decreased *c-Myc* expression and vice versa, while *miR-155* inhibitors increased *c-Myc* expression (Figure 5A). Then, we examined whether *c-Myc* was involved in the regulatory effect of SM on cells. The results obtained from qRT-PCR assay showed that the expression of *c-Myc* decreased by 71.66% after 5 μM SM treatment for 48 h, and the *miR-155* inhibitor was able to significantly weaken the inhibitory effect of SM on *c-Myc* expression (Figure 5B). Immunoblotting assay verified that SM (2 and 5 μM) downregulated c-Myc protein expression and upregulated P53 protein (Figures 5C,D). Taken together, these results suggested that SM downregulated lncRNA *HOXA11-AS,* which in turn regulated *c-Myc* gene expression through sponge *miR-155*.

### SM Inhibits FaDU Development *In Vivo*

**FIGURE 6:**
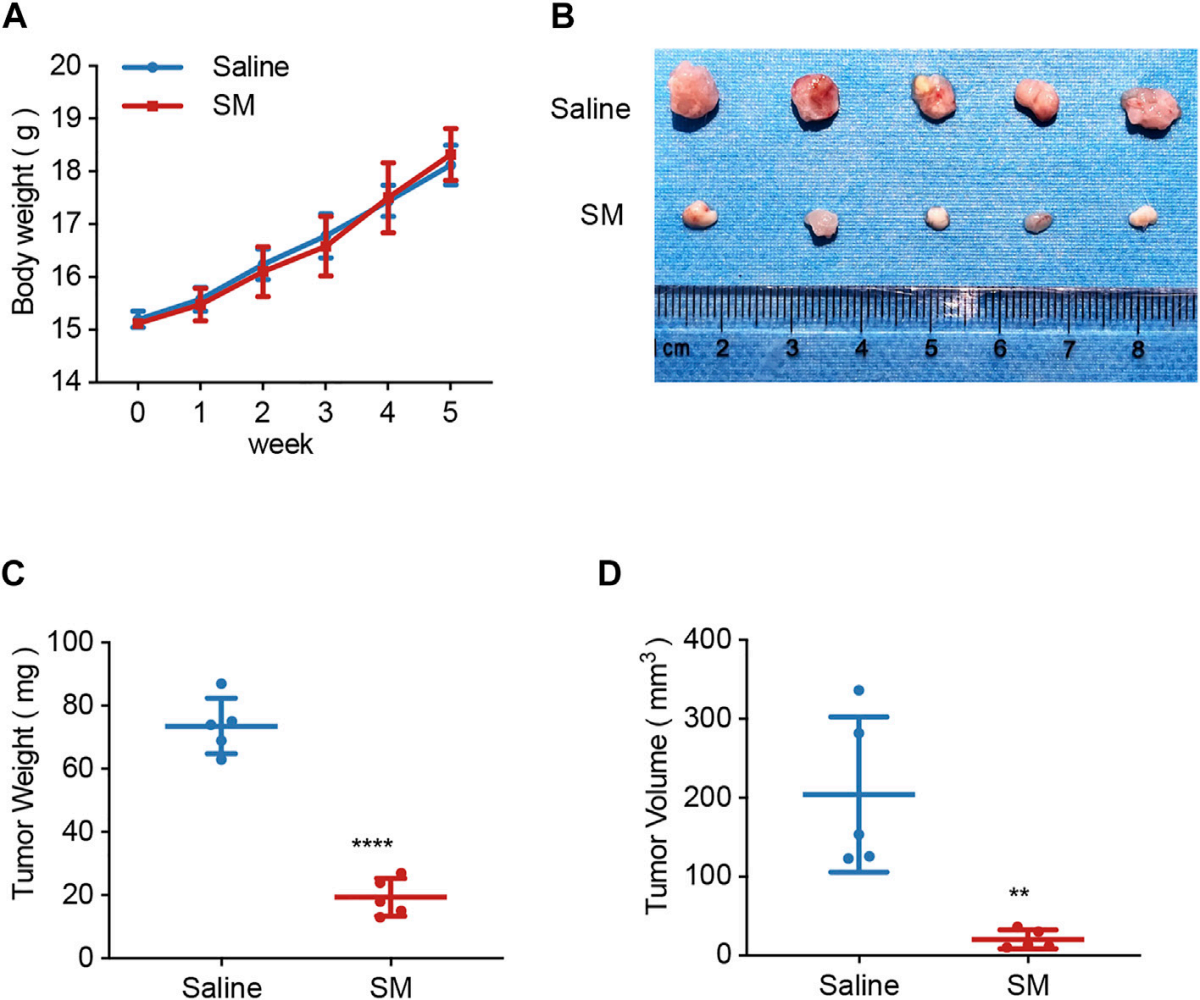
SM inhibits FaDU xenograft tumor development *in vivo*. **(A)** Mice in the SM group were injected with SM (10 mg/kg, i. v.) once daily, and the mice that were injected with saline served as controls. The body weights of the mice were recorded weekly. After 5 weeks, tumors were separated, and their volume and weight were recorded. **(B)** Xenograft tumor morphology of the saline group and SM group. **(C)** Tumor weight of the saline group and SM group. **(D)** Tumor volume of the saline group and SM group. ^**^(*p* < 0.01) and ^****^(*p* < 0.0001) indicate statistically significant differences.

To investigate the tumor growth inhibitory effect of SM *in vivo*, a mouse xenograft tumor model was established, in which FaDU cells (2 × 10^6^) were injected subcutaneously into the right axillary fossa of six-week-old female BALB/c mice. One week later, SM or saline was administrated. The mice in the SM group were injected with SM (10 mg/kg, i.v.) once daily, and the mice that were injected with saline served as controls. Body weights of the mice were recorded weekly. After 5 weeks of administration, tumors were separated, and their volume and weight were recorded. The data showed that there was no significant difference in body weight between the SM group and control, suggesting that there was no obvious toxicity in SM-treated mice (Figure 6A). The tumor volume and tumor weight decreased successively from 204.10 ± 80.01 mm^3^ to 20.53 ± 10.80 mm^3^ and from 73.6 ± 7.94 mg to 19.4 ± 5.31 mg, respectively, by SM treatment for 5 weeks, indicating that SM exerted a strong inhibitory effect on tumor development *in vivo* (Figures 6B–D).

## Discussion

HSCC is one of the head and neck cancers with a low incidence rate, but the prognosis is poor (Kwon and Miles, 2019). As its early stage is not easy to be detected, most patients are at an advanced stage, which increases the difficulty of treatment (Kuo et al., 2014). Although surgical treatment is capable of gaining sound local control, it will affect the normal speech function of patients and reduce their quality of life (Pracy et al., 2016; Wu et al., 2021). Chemotherapy remains one of the main strategies for HSCC, mainly including cisplatin (Janoray et al., 2020). The challenges of HSCC treatment necessitate the urgent search for a chemotherapeutic agent with few side effects and notable efficacy for patients.

LncRNAs were previously considered a byproduct of the transcription process, but with the in-depth into their research (Ma et al., 2013), their essential role in the development of many diseases becomes increasingly recognized, especially in cancer research. The aberrant expression or mutation of lncRNAs participates in a variety of tumor pathogenesis mechanisms, which include the regulation of proliferation, apoptosis, invasion, and migration of tumor cells (Xu et al., 2014; Bhan et al., 2017). One study of lncRNA and mRNA expression profiles in patients with HSCC identified differential expression of lncRNAs and mRNAs in cancer compared to para-cancer tissues, suggesting a potential regulatory role for multiple lncRNAs during the development of HSCC (Wang et al., 2016; Zhang et al., 2021).

We observed that SM exhibited a strong killing effect on HSCC cells, FaDU. The inhibitory effect of SM on tumor development was verified by establishing a xenograft tumor model. Then, high-throughput sequencing data clarified a key target of SM to inhibit cell proliferation, lncRNA *HOXA11-AS*. We verified that SM downregulated lncRNA *HOXA11-AS*, which could sponge *miR-155*, in turn, inhibited *c-Myc* and *P53* expression, resulting in suppression of cell proliferation inhibition and apoptosis. *P53* is a classic tumor suppressor gene, and *MYC* is the best-characterized proto-oncogene. The proto oncoprotein *c-Myc* and the tumor suppressor *P53* are inextricably linked in regulating cell survival and proliferation (Huang et al., 2021). Therefore, we examined the protein expression of P53 in this study to observe the anticancer effect of SM more comprehensively.

*HOXA11-AS* is the antisense lncRNA of *HOXA11* that regulates the proliferation and invasion of cancer cells (Xue et al., 2018). MicroRNA *miR-155* exerts significant functions in physiological processes such as cell cycle, apoptosis, and proliferation, which are involved in regulating the development of multiple cancers (Yang et al., 2018; Michaille et al., 2019). Liu et al. demonstrated that *miR-155* directly targeted programmed cell death 4 (PDCD4) to promote lung cancer cell growth in a model of nude mice (Xia and Zhao, 2020). Xu et al. (2020) pointed out that as a molecular sponge for *miR-155*, *HOXA11-AS* was significantly upregulated in HSCC and exerted a pro-tumorigenic effect by sponging a tumor suppressor *miR-155* in HSCC. Our results indicated that SM was able to inhibit the development of FaDU xenograft tumors through suppression of HOXA11-AS, also providing evidence for *HOXA11-AS* on tumor regulation.

There is no direct experimental evidence about the molecular mechanism responsible for SM-induced *HOXA11-AS* downregulation in HSCC yet. Recently, growing evidence proves that transcription factors play a vital role in up- or downregulated lncRNAs in multiple cancers (Chen et al., 2019; Shuai et al., 2020). One study found that transcription factor homeobox B13 (*HOXB13*), identified as an upstream regulator of *HOXA11-AS*, could positively regulate the expression of *HOXA11-AS* in prostate cancer. Also, the *HOXA11-AS* combination with *HOXB13* manipulates the level of bone-specific metastasis-related genes of prostate cancer (Misawa et al., 2021). SM regulates multiple signaling pathways related to tumor progression and drug resistance, such as tumor suppressor pathways, mitochondrial pathways, and death receptor pathways (Wu et al., 2020; Tang et al., 2022). In this study, SM induces apoptosis via the lncRNA *HOXA11-AS/miR-155/c-Myc/p53* pathway. Therefore, a potential mechanism for SM downregulating *HOXA11-AS* expression is that SM may activate the upstream transcription factors of *HOXA11-AS* at the transcriptional level.

Natural compounds provide vital resources for the development of antitumor agents (Ijaz et al., 2018). Solamargine (SM), the extract of *Solanum nigrum*, exhibits a variety of therapeutic activities (Kalalinia and Karimi-Sani, 2017). Dozens of studies have demonstrated its potent killing effect on a variety of cancer cell lines. It is worth mentioning that SM shows an extremely low IC_50_ compared to most natural compounds. Liang et al. (2004) showed that the IC_50_ of SM on A549 cells was 2.9 μM, and Kuo et al. (2000) showed that the IC_50_ of SM on Hep3B cells was 5 μg/ml. We demonstrated for the first time the killing effect of SM on FaDU cells with an IC_50_ of 5.17 μM, which is comparable to other cancer cell lines, suggesting that SM has no obvious significantly selective killing effect on tumor cells. Considering the safety profile, Al Sinani et al. (2016) demonstrated that the cytotoxic effect of SM on human cancer cells was selective, evidenced by that it was able to selectively kill rapidly proliferating tumor cells with less damage to normal cells, indicating the promise of SM as an antitumor agent with few side effects and robust tumor-killing activity. The main mechanisms by which SM kills tumors include apoptosis, cycle arrest, and inhibition of tumor cell migration and invasion (Kalalinia and Karimi-Sani, 2017). Although the antitumor effect of SM has been widely recognized, the study of its effect on HSCC therapy is still blank. Our study confirmed the anti-HSCC tumor efficacy of SM for the first time, which revealed and broadened its application. We also explored the mechanism of SM killing FaDU cells with regard to ncRNA.

## Conclusion

In this study, we revealed the effect of SM on FaDU cell phenotypes. According to the vital role of lncRNA *HOXA11-AS* in HSCC and our data obtained from high-throughput sequencing, we revealed the involvement of *HOXA11-AS* in the inhibitory effect of SM on the proliferation of FaDU cells. In conclusion, SM could be used in the development of an anticancer compound for HSCC, which has great prospects in the future.

## Data Availability

The original contributions presented in the study are publicly available. These data can be found at: https://www.ncbi.nlm.nih.gov/sra, PRJNA821688
