# Supplementary material for: Solamargine Inhibits the Development of Hypopharyngeal Squamous Cell Carcinoma by Decreasing LncRNA HOXA11-As Expression
Source: Front Pharmacol. 2022 Jul 12;13:887387. doi: 10.3389/fphar.2022.887387 (PMC9315292; doi:10.3389/fphar.2022.887387)
Supplement: Supplementary file 1 [file DataSheet1.PDF]

Supplementary Information to

Solamargine Inhibits the Development of Hypopharyngeal Squamous Cell Carcinoma by Decreasing LncRNA *HOXA11-As* Expression

Ying meng[1†], Mengli Jin [1†], Dai yuan [1†], Yicheng Zhao [3,4], Xiangri Kong [1], Xuerui Guo [2], Xingye Wang [1], Juan Hou [1], Bingmei Wang [1*], Wu Song [1*] and YongTang [1*]

****Correspondence:***
Bingmei Wang: bingmeiwang1970@163.com
Wu Song: five841110@126.com
Yong Tang: 15943012058@163.com
† These authors have contributed equally to this work

S1

A

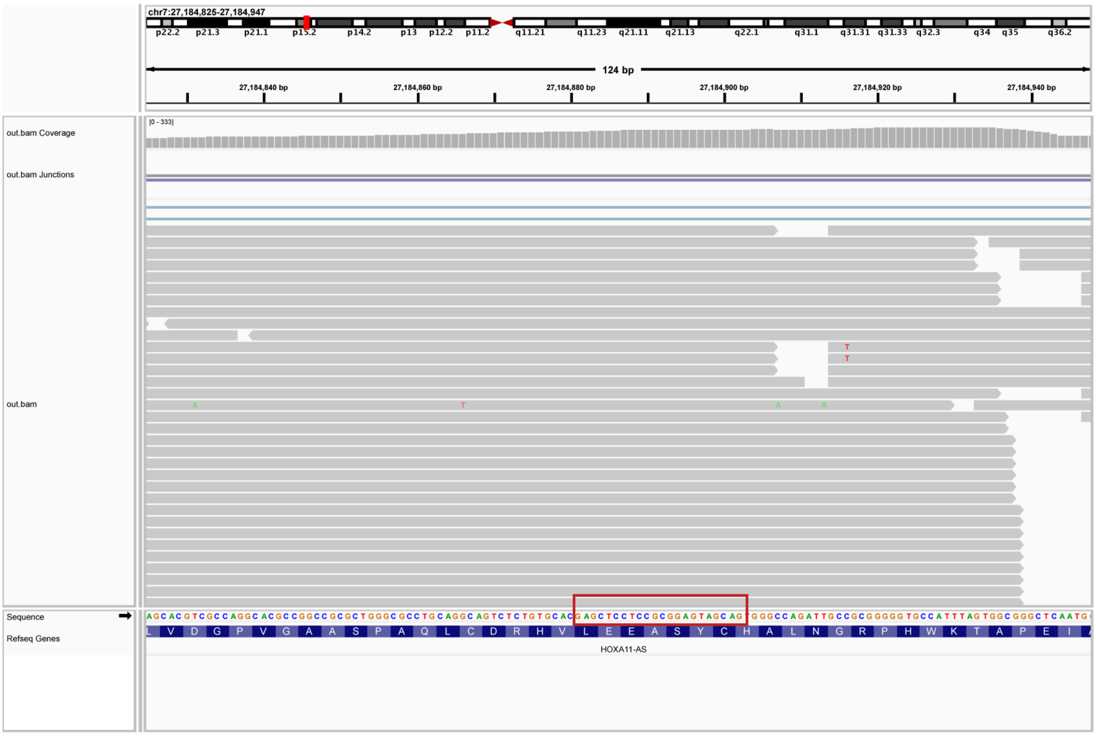

B

c-Myc-WT: 5'-aaUCCUAGUAU-AUGUACCUAGUAUUA 3'
miR-155: 3'-ugGGGAUAGUGCUA------AUCGUAAU 5'
c-Myc-MUT: 5'- aaUCCUAGUAU-AUGUACCCGACGCCG 3'

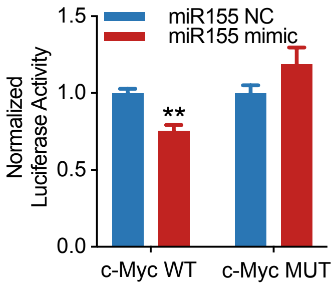

Fig.S1 A) The wiggle plot of lncRNA HOXA11-AS. Sequence within the red box indicate binding site for miR-155 on HOXA11-AS sequences. **B)** Relative Luciferase activity of 293 cells co-transfected with *miR-155* NC or *miR-155* mimics and the wild type or mutated *c-Myc*.
